# Translation and Validation of the Short Form of the Fear of Dental Pain Questionnaire in China

**DOI:** 10.3389/fpsyg.2021.721670

**Published:** 2021-11-23

**Authors:** Lei Wu, Heather Buchanan, Arjen J. van Wijk

**Affiliations:** ^1^Key Research Base of Humanities and Social Sciences of the Ministry of Education, Tianjin Normal University, Academy of Psychology and Behavior, Tianjin, China; ^2^Medical School, University of Nottingham, Nottingham, United Kingdom; ^3^Department of Social Dentistry and Behavioural Sciences, Academic Centre for Dentistry Amsterdam (ACTA), University of Amsterdam, Amsterdam, Netherlands

**Keywords:** anxiety, pain, questionnaire, China, survey

## Abstract

The short form of the Fear of Dental Pain Questionnaire (s-FDPQ) is a validated measure developed to screen patients for their fear of pain associated with dental procedures. As there is a high prevalence of dental fear/anxiety in Chinese adults, the primary aim of our study was to translate the s-FDPQ into standard Mandarin and explore its reliability and validity with Chinese adults. The second aim of our study was to explore fear of dental pain (FDP) scores in relation to dental attendance, anxiety and gender. We translated the s-FDPQ using the forward-backward method. It was completed by 480 Chinese adults alongside the Modified Dental Anxiety Scale (MDAS; Chinese version) to test convergent validity. 109 participants completed the s-FDPQ again 14 days later to evaluate test-retest reliability. The Chinese s-FDPQ (s-CFDPQ) was internally consistent (alpha = 0.87) and demonstrated convergent validity (*r* = 0.73 when correlated with the MDAS). Test-retest reliability was good (ICC = 0.86). Individuals who had never attended the dentist (22%) had higher FDP scores than those that had, even if they were not dentally anxious. Also, females reported higher FDP scores (*p* < 0.001). These findings suggest that the s-CFDPQ is a reliable and valid measure for assessing fear of dental pain in Chinese adults. The s-CFDPQ could allow quick identification of individuals who are fearful of dental pain who may require specialist attention.

## Introduction

China is a developing country whose population accounts for one fifth of the world’s population. Despite rapid economic development, oral health care has traditionally been a neglected area in terms of the nation’s health (Zhou et al., 2018) with dentistry seen as a separate speciality from general medicine. The Chinese Government provides basic medical insurance but this does not cover preventative oral health services. Thus, dental visits tend to be treatment-oriented, which means that people do not seek dental care unless they have acute symptoms, such as severe toothache or periodontal symptoms (Liu L. et al., 2015). Dental attendance rates are low with 30% of respondents in the “2019 New Era and Living Conditions in Megacities Survey” (Qi et al., 2021) (NELCMS) reporting that they had never attended the dentist.

There is also poor engagement in oral self-care practices. The latest National Oral Health Epidemiological Study (NEOH) showed that less than half of Chinese adults brush their teeth twice a day (Si et al., 2019). Moreover, nationally the rates of dental disease are concerning. The caries rate exceeds 50% (Si et al., 2019) and the prevalence of periodontitis is very high, with 90% of adults in Mainland China suffering from periodontal disease of varying severity (Jiao et al., 2021). Compared with 10 years ago, periodontal rates have not improved (Liu et al., 2016) with some parameters worse than they were.

The national Government in China has recognised the need for change, and oral health as a public health concern is now on the national Agenda. The Healthy China 2030 blueprint has been released which sets out oral health as a priority within the all-round development of a healthy China (The State Council of China, 2017), highlighting the commitment to investing in public oral health and the need to move from treatment-focused to prevention-centred practices.

This projected change in oral health care is a positive step forward in China. However, as outlined above there are currently very high levels of dental disease. Moreover, individuals tend to attend only when suffering from painful symptoms, thus increasing the likelihood of invasive and painful treatment. This situation may mean that individuals who attend the dentist are anxious and fearful of pain. Indeed, those who have never been to the dentist may also be anxious and fearful of pain—from hearing of others’ bad experiences, negative observations of dentistry or how dentistry is portrayed in the media (Locker et al., 1999; Appukuttan et al., 2015).

Compared to research in the West, there is much less research which provides prevalence data on dental anxiety in China. Research that has been conducted using validated dental anxiety scales have shown that dental anxiety is prevalent in China. For example, Liu Y. et al. (2015) in a sample of 1,203 Chinese patients found that 23.4% of the sample were highly fearful according to their scores on the Dental Anxiety Scale (Corah, 1969). The authors highlight this is notably higher than other Western countries, such as the United Kingdom and Australia.

Fear and anxiety have an important role in the experience of both acute and chronic pain (Hirsh et al., 2008; Zaman et al., 2015). For example, patients with high dental anxiety are likely to have exaggerated memory and prediction of dental pain. Research with Western dental patients support the concept of a cycle of anxiety and exaggerated expectation and recall of pain. For example, in a United Kingdom general dental practice study, all patients recalled more pain after 3 months than they reported immediately after the dental procedure. However, highly dentally anxious patients had a greater increase in recalled pain (Kent, 1985). Moreover, it has been shown that patients with high dental anxiety undergoing tooth extraction have exaggerated memory and prediction of both and dental pain and anxiety (McNeil et al., 2011).

Fear of pain and pain intensity in pain patients, has also been frequently investigated with a recent meta-analysis confirming that fear of pain contributes to increased pain perceptions (Markfelder and Pauli, 2020). For dental patients, fear of dental pain (FDP) relates to both pain experienced and anxiety. For example, van Wijk and Hoogstraten (2005) found that FDP was related to pain experienced during periodontal probing. Moreover, within their sample of very anxious dental patients a decrease in dental anxiety was associated with lower FDP scores. This suggests that FDP is partly dependent on the level of dental anxiety. However, though anxiety and FDP are related, they are still separate constructs. Indeed, Lin et al. (2017) recommend that both fear and anxiety should be assessed in relation to the dental context, as they are different experiences with divergent effects on pain.

### Assessing Fear of Dental Pain

The Fear of Dental Pain Questionnaire (FDPQ) (van Wijk and Hoogstraten, 2003) is an 18-item self-report scale assessing FDP associated with a variety of dental procedures. The authors state that it is based on the theory that anxious people expect more pain, that expecting more pain can then make the individual anxious, and anxious people may feel pain more strongly, as they focus their attention on the painful stimulus. Therefore, for individuals who react fearfully to pain, they may get caught in a vicious cycle of overestimated pain and increasing anxiety.

Besides the 18-item version of the FDPQ, there is also a briefer 5-item version developed to more efficiently screen patients for research and practice. In a validation study (van Wijk et al., 2006), items retained on the s-FDPQ (short-form) include fear of pain associated with receiving an anaesthetic injection in the mouth, root canal treatment and having a tooth extracted. In the same study, the s-FDPQ correlated well with the original 18 item version and demonstrated sound psychometric properties.

Comprehensive assessment of psychological variables, such as fear of pain rely on accurate screening, using an instrument that is relatively brief, accurate and accessible to a variety of patient and non-clinical populations (McNeil et al., 2018). It would be valuable to have such a fear of pain measure available in Chinese in order to explore this concept in relation to dental procedures for both clinical and research purposes. Therefore, the primary aim of this study is to translate the s-FDPQ into standard Mandarin and explore the reliability and validity of this Chinese version with native Chinese adults. Translation and validation should be carried out before using a measure in another culture or population. Thus, we carried this out in two-phases. First, we generated a semantically equivalent translation and assessed the face validity and coherence/understanding of items and instructions of the Chinese short form of the Fear of Dental Pain Questionnaire (s-CFDPQ). We then followed this by the assessing the psychometric properties of the measure.

The second aim of our study was to explore FDP scores in terms of dental attendance, anxiety and gender. There is a paucity of data on non-attenders in dental care, therefore we wanted to explore whether there was a difference in total s-FDPQ scores between those who had never been a dental patient, and those who had. Second, to add to the knowledge on how dental anxiety and FDP are related, we compared very dentally anxious non-attenders, with non-attenders who were not highly dentally anxious and regular attenders (who were not highly dentally anxious). In addition, we investigated whether there was a gender difference in FDP scores. Findings from European and American studies have tended to show that women report more fear of pain than men (McNeil and Rainwater, 1998; Albaret et al., 2004; Roelofs et al., 2005; Di Tella et al., 2019) but there may also be cultural factors that influence this relationship. Therefore, it would be interesting to test this in our Chinese sample.

## Materials and Methods

### Phase 1: Forward-Backward Translation of the Short Fear of Dental Pain Questionnaire

The short Fear of Dental Pain Questionnaire (s-FDPQ) was translated following the forward-backward translation method. Firstly, the primary author who is a Chinese National and is bilingual (first language Mandarin but fluent in English) translated the s-FDPQ into Mandarin. Secondly, another bilingual Chinese National (who was not involved in the research and had not accessed the original s-FDPQ), conducted a back translation. The third step was to compare the original English version and the back-translated version. A panel was convened of two psychologists, who are researchers in psychology and dentistry, and the primary author (also a psychologist). One of the panel members was familiar with the s-FDPQ, the other co-authored the validation study of the English version of the s-FDPQ. Following discussion, some minor changes were made.

A pilot study was then conducted with 35 participants (21 females) living in Tianjin, North China. The participants fulfilled the inclusion criteria of being 18 years or older with basic literacy and reading ability. They were asked to complete the s-FDPQ and comment on the language, understanding and ease/difficulty of completion. We also asked that they express their opinion on the face validity of the items. As with the English validation study, face validity was based on whether each of the items describe a common, potentially painful, dental procedure. The rationale being that most participants need to have either experienced a dental procedure, or know about it to such an extent that they have expectations of what it would involve. Minor changes to wording were made by the first author and this was checked again by the two psychologists. No more changes were made at this point.

### Phase 2: Psychometric Properties of the Short Fear of Dental Pain Questionnaire

#### Participants and Procedure

Participants were a convenience sample of 480 adults (325 females); 138 were recruited from Tianjin city in North China, 169 from Dongying city in Shandong province (North China) and 173 from Wuhan city in Hubei province (South China).

The study was advertised across a variety of different communities and colleges, and as an incentive to take part participants were gifted a pen or fan. To take part in the study participants needed to be 18 years or above and have basic literacy and reading ability. Interested participants were informed that the study was investigating how people feel about going to the dentist, and the different procedures and treatments they may potentially have there. They were told that the questionnaire was anonymous and confidential. If they agreed to take part, they were then given a questionnaire pack to complete. In order to evaluate test-retest reliability, participants were also asked for an email address if they would be willing to complete the s-FDPQ gain 14 days later. From the 130 who gave their email address, 109 (74 females) completed the s-FDPQ.

Ethical approval was granted from the Ethics Committee of Tianjin Normal University (APB20161205). All participants gave written informed consent in accordance with the Declaration of Helsinki.

#### Materials

The paper-based questionnaire pack began with questions on demographics (such as City/Province, gender, age, and occupation), dental attendance and dental procedures encountered (e.g., having a tooth filled) and items related to oral self-care (e.g., tooth-brushing) (see Table 1). Following this, they were asked to complete the s-FDPQ which begins (as with the English version) with the instruction: Participants are asked to look at each item carefully. They are then asked to “Think about how FEARFUL you are of experiencing the PAIN associated with each item. If you have never experienced the PAIN of a particular item, please answer on the basis of how FEARFUL you expect you would be if you had such an experience. Circle one number per item to rate your FEAR OF PAIN in relation to each event.” Items were: receiving an anaesthetic injection in the mouth; having a tooth drilled; receiving root canal treatment; having a tooth pulled and having a wisdom tooth extracted. Responses are on a 5-point Likert-type scale, ranging from 1 = no fear to 5 = extreme fear. Total scores range from 5 to 25.

**TABLE 1:** Demographics and dental health history and habits of main study participants.

Participants (*N* = 480)		Frequency (%)
Gender	Male	155 (32.29)
	Female	325 (67.71)
Age categories	Below 20 years	82 (17.08)
	20–30 years	210 (43.75)
	31–40 years	69 (14.37)
	41–50 years	55 (11.46)
	Over 50 years	64 (13.33)
Province	Urban	345 (71.88)
	Rural	135 (28.13)
Highest education	Middle school or below	59 (12.29)
	High school	86 (17.92)
	College/University undergraduate	243 (50.63)
	University postgraduate	92 (19.17)
Occupation	Civil service and public institution employees	83 (17.29)
	Enterprise/company employees	97 (20.21)
	Self-employed	36 (7.50)
	Farmer	33 (6.88)
	College student	183 (38.13)
	Not working	48 (10)
Monthly income (Yuan)	Low <2000	266 (55.4)
	Middle 2,000–10,000	179 (37.3)
	High >10,000	35 (7.3)
How often do you brush teeth?	Once a day	142 (29.58)
	Twice a day	289 (60.21)
	More than twice a day	29 (6.04)
	Not regularly	14 (2.92)
	Not at all	6 (1.25)
Have you ever attended a dentist?	Yes	373 (77.7)
	No	107 (22.3)
Frequency of dentist visits	Twice a year	44 (9.17)
	Once a year	80 (16.67)
	Less than once in 2 years	239 (49.79)
	Other answer	117 (24.38)
What kind of dental procedures have you experienced?	Dental check-up	161/480 (33.54)
	Tooth extraction	196/480 (40.83)
	Scale and polish (“Tooth wash”)	128/480 (26.67)
	Orthodontics	56/480 (11.67)
	Tooth filled	168/480 (35.00)
	Local anaesthetic injection	78/480 (16.25)
	Root canal therapy	54/480 (11.25)
	Conscious-sedation	10/480 (2.08)
	Other	44/480 (9.17)

One of the aims of our study was to test the validity of the newly translated s-FDPQ. In order to test convergent validity, we included the Chinese Modified Dental Anxiety Scale (MDAS; Yuan et al., 2008) in the questionnaire pack. This five-item scale was originally developed in English (Humphris et al., 1995) and items relate to dental procedures and the dental context more generally (e.g., “If you were about to have a tooth drilled, how would you feel?”). Responses are on a 5-point Likert-type scale from 1 = Not anxious to 5 = Extremely anxious. Total scores range from 5 to 25 with a score of 19 indicating a participant is “very dentally anxious” (Humphris et al., 1995; King and Humphris, 2010). The Chinese MDAS has demonstrated sound psychometric properties (Yuan et al., 2008). We would expect a moderate to strong correlation between fear of dental pain and dental anxiety as findings suggest that fear of dental pain is partly dependent on level of dental anxiety (van Wijk and Hoogstraten, 2003).

### Statistical Analyses

We used Cronbach’s alpha to test the internal consistency of the s-FDPQ and item-total correlations to explore whether the s-FDPQ measures a single underlying construct. We performed an intra-class correlation coefficient (ICC) between the first and second administration of the s-FDPQ for the sub-group of participants who completed the s-FDPQ 14 days apart. The convergent validity of the scale was assessed by correlating the s-FDPQ with the Modified Dental Anxiety Scale. In addition, an independent-samples t-test was used to explore whether there was a difference in total s-FDPQ scores between (1) those who had never been a dental patient, and those who had and (2) males and females. We conducted a one-way ANOVA to compare very dentally anxious non-attenders (those scoring 19 and above on the MDAS), with non-attenders who were not highly dentally anxious (scoring less than 19 on the MDAS) and regular attenders (who were not highly dentally anxious; <19 on the MDAS). A repeated-measures analysis of variance (ANOVA) was employed to establish if there was a difference in s-FDPQ scores across items.

## Results

There were 530 questionnaires given out, and 516 questionnaires returned. If there were any missing responses to any items on the MDAS or s-FDPQ, we did not include the questionnaire in the analysis, leaving 480 completed questionnaires (325 females). Details of participants are presented in Table 1. Most participants were younger and lived in urban areas. There were 22% of the participants who had never been a dental patient. Having a tooth pulled was the dental procedure that most participants had experienced, with only 16% of the sample having experienced a local anaesthetic injection. Tooth-brushing (at least) twice a day was reported by most of participants, though a third of participants reported less frequent brushing.

There was a high level of internal consistency for the Chinese s-FDPQ (α = 0.87) and all corrected inter-item correlations (see Table 2) showed an acceptable range (0.63–0.71). There was also a strong intra-class correlation (ICC = 0.86) between the first and second responses for the 109 participants who completed it 14 days apart.

**TABLE 2:** Mean scores (SD) and corrected item-total correlations (CITC) for each s-FDPQ item.

	Mean	SD	CITC
Total score (5 items)	14.6	4.5	
1. Receiving an anaesthetic injection in the mouth	2.4	1.0	0.63*
2. Having a tooth drilled	3.0	1.1	0.70*
3. Receiving root canal treatment	3.1	1.1	0.68*
4. Having a tooth pulled	2.9	1.2	0.71*
5. Having a wisdom tooth extracted	3.1	1.2	0.66*

*p < 0.001.

The Chinese Modified Dental Anxiety Scale was internally consistent in our sample (α = 0.85). The mean score was 13.7 (*SD* = 4.6) with 75 participants (15.63%) scoring 19 or above, indicating they are “very dentally anxious.” There was a strong significant correlation (*r* = 0.73, *p* < 0.001) between the s-FDPQ and the MDAS, demonstrating convergent validity.

Means and standard deviations (SD) for the total (and each of the items) of the s-FDPQ are in Table 2. The s-FDPQ mean score was 14.6 (*SD* = 4.5) ranging from 5 to 25. To test whether there was a difference in fear of dental pain across items, we employed a repeated-measures ANOVA. There was a significant within-subjects effect [*F*(4, 475) = 30.8, *p* < 0.01]. Pairwise comparisons showed that the mean item score was significantly lower for the local anaesthetic injection than for any of the other items (*p* < 0.001). Also, the mean item score was significantly lower for having a tooth pulled compared to the items about root canal treatment and having a wisdom tooth extracted (*p* < 0.001).

Females scored significantly higher (mean = 15.25; *SD* = 4.41) than males (mean = 13.09, *SD* = 4.36) on the s-FDPQ indicating females are more fearful of dental pain [*t*(478) = 5.04, *p* < 0.01]. Those who had never attended the dentist had a mean FDPQ-s score of 16.05 (*SD* = 4.75) and those that had previously attended scored a mean of 14.13 (*SD* = 4.34). When we compared these there was a significant difference [*t*(478) = 3.95, *p* < 0.01] with those having never attended having higher fear of dental pain scores. There was also a significant difference in FDP scores for those with different levels of attendance and anxiety (see Table 3) [*F*(2, 12) = 132.52, *p* < 0.001]. *Post hoc* tests showed that highly anxious non-attenders had significantly higher FDP scores than non-anxious non-attenders and non-anxious regular attenders (*p* < 0.001). Non-attenders there were not highly anxious had significantly higher FDP than regular attenders who were not highly dentally anxious (*p* < 0.001).

**TABLE 3:** Fear of dental pain (FDP) scores in relation to dental anxiety and attendance.

Group	N	Mean (SD) FDP score
Non-attenders (not highly dentally anxious)	81	14.64 (2.96)
Non-attenders (with high dental anxiety)	26	21.19 (1.65)
Regular attenders (not highly dentally anxious)	122	10.89 3.33

## Discussion

In summary, our main findings demonstrated good psychometric properties for the Chinese version of the short form of the Fear of Dental Pain Questionnaire (s-CFDPQ). The scale demonstrated a high level of internal consistency, especially considering the s-CFDPQ has only five items. The Cronbach’s Alpha directly compares to that in the original validation of the English s-FDPQ (van Wijk et al., 2006)As predicted there was a moderately strong correlation between the Modified Dental Anxiety Scale (MDAS) and the s-CFDPQ which demonstrates good convergent validity. This also shows that dental anxiety and FDP are related, but also separate constructs, and as such should be measured separately. Indeed, Lin et al. (2017) suggest that both fear of pain and anxiety should be assessed in relation to the dental context. Moreover, we found that females reported higher FDP scores than males, which is similar to European and American studies on fear of pain (McNeil and Rainwater, 1998; Albaret et al., 2004; Roelofs et al., 2005; Di Tella et al., 2019). However, we do not know if this difference reflects a tendency for females to respond more fearfully to pain, or whether females are more comfortable reporting fear of pain.

There was a high number of participants (22%) that had never been to a dentist. This is similar to previous findings exploring dental service utilisation in China. For example, 30% of respondents in the “2019 New Era and Living Conditions in Megacities Survey” reported that they had never attended the dentist (Qi et al., 2021). It is interesting that so many individuals in our sample have never experienced the specific procedures included in the scale, yet still report a considerable level of FDP. Indeed, the participants who had never attended the dentist had higher FDP scores than those who had. It may be that this is related to individuals overestimating fear of pain for procedures (and pain) they had not experienced. For example, van Wijk and Hoogstraten (2005) found that participants tended to overestimate their FDP when they have not actually experienced the specific pain itself. We also found those with high anxiety who have never been to the dentist appear to have the highest FDP scores (compared to non-anxious never-attenders and regular attenders). However, even among those individuals who were not dentally anxious, never-attenders had significantly higher FDP than regular attenders. Hearing of others’ bad experiences, negative observations of dentistry (from friends/family) or how dentistry is portrayed in the media may contribute to high levels of FDP in never-attenders. Thus, targeting FDP may be especially important for those individuals who have never been a dental patient.

Having a tooth pulled was the dental procedure that most participants had experienced (40%), with only 16% of the sample having experienced a local anaesthetic (LA) injection. This indicates a less than optimal use of local anaesthesia in Chinese dental care. This may be related to culture. For example, Moore et al. (Moore et al., 1998) explored ethnic differences of pain and the need for LA for tooth drilling across Anglo-American, Mandarin Chinese, and Scandinavian dentists. American dentists reported that about 1% of their adult patients did not use LA compared with 90% among Chinese patients. Chinese dentists made their decisions not to use anaesthetics because they explained drilling as only a “sour” sensation, whereas injections were described as “painful.” It has also been noted that communication between dentists and patients is “one way” in China, with dentists making the decision as to when anaesthesia is appropriate (Domoto et al., 1990). This may be related to FDP (and also experienced pain for those who have been a dental patient) in our study. Prevention of dental pain (including use of LA) should make for a better patient experience, as would a more patient-centered approach from dentists. Positive experiences may then be observed by these non-attenders in terms of family/friends (and the media) which may help encourage those who have not been a dental patient to attend.

It is important to recognise the contribution this preliminary study makes to the literature and the strengths of the study. First, this is the first translation, and validation of the Fear of Dental Pain Questionnaire (short-form) in Chinese. Second, there is a paucity of data comparing never-attenders with those who have been a dental patient in terms of FDP and dental anxiety. Our study provides valuable preliminary data comparing these two groups on key psychological variables.

It is also important to acknowledge the limitations of the study. First, we could have included a Chinese dentist in the study team, which may have provided us with clinical insights (e.g., in the use of local anaesthesia in China). Second, although we sampled from different provinces, we cannot claim to generalise our findings across China. Third, the test-retest period was 2 weeks in order to maximise the number of individuals who completed the questionnaire for a second time. However, in doing so we acknowledge that participants may have had more opportunity to remember their scores from the initial completion. Finally, there were significantly more females in our sample. It should be noted, however, that females do appear more willing to take part in studies on FDP, and as such there is an over-representation of females in study samples across both clinical and non-clinical settings (van Wijk and Hoogstraten, 2003, 2005; van Wijk et al., 2006).

We have identified future directions and recommendations from our study findings. First, a key important question to consider is—would we recommend using the s-CFDPQ when many individuals have no experience of the dental procedures included in the scale? We checked the face validity of the s-CFDPQ in the first phase of the study, and participants who had never been a dental patient knew enough about the procedures to have expectations of what they would involve. Therefore, we feel confident that participants are rating their FDP on procedures they recognise, and as such we would recommend using this scale in further studies in China. Indeed, the s-CFDPQ could be used in a large-scale longitudinal study which compares attenders and never-attenders, we would suggest including not only FDP and dental anxiety, but also pain experienced and use of LA. Patients could then be followed up to capture memory of both pain and anxiety. In addition, the s-CFDPQ could be used in clinical practice to help dentists identify patients who are very fearful of pain. These patients may require special attention, longer appointments, and specific dental pain management (van Wijk et al., 2006). In addition, the s-CFDPQ could help evaluate the effectiveness of psychological interventions (for example, cognitive behavioural therapy) for highly fearful patients. Finally, it would be valuable to explore how FDP has evolved for highly fearful never-attenders. Ensuring they have a positive experience if they do become a dental patient, is also an important endeavour.

## Conclusion

In conclusion, this is the first translation, and subsequent validation, of the Fear of Dental Pain Questionnaire (short-form) in Chinese. The s-CFDPQ could be used in future studies exploring FDP, in both attenders and never-attenders. It may also allow dentists to quickly identify patients who may require specialist attention, and possibly psychological intervention such as cognitive behavioural therapy.

## Data Availability Statement

The raw data supporting the conclusions of this article will be made available by the authors, without undue reservation.

## Ethics Statement

The studies involving human participants were reviewed and approved by the Ethics Committee of Tianjin Normal University (APB20161205). The patients/participants provided their written informed consent to participate in this study.

## Author Contributions

HB conceived the idea of the study. HB and LW designed the study. LW collected and analysed the data. HB, AW, and LW co-wrote the manuscript. All authors read and approved the final manuscript.

## Conflict of Interest

The authors declare that the research was conducted in the absence of any commercial or financial relationships that could be construed as a potential conflict of interest.

## Publisher’s Note

All claims expressed in this article are solely those of the authors and do not necessarily represent those of their affiliated organizations, or those of the publisher, the editors and the reviewers. Any product that may be evaluated in this article, or claim that may be made by its manufacturer, is not guaranteed or endorsed by the publisher.
